# Aspirin Use and Risk of Hepatocellular Carcinoma in a National Cohort Study of Korean Adults

**DOI:** 10.1038/s41598-018-23343-0

**Published:** 2018-03-21

**Authors:** In Cheol Hwang, Jooyoung Chang, Kyuwoong Kim, Sang Min Park

**Affiliations:** 1 Department of Family Medicine, Gachon University Gil Medical Center, Incheon, 405-760 Republic of Korea; 2 Department of Biomedical Sciences, Seoul National University Graduate School, Seoul, 03080 Republic of Korea; 3 Department of Family Medicine, Seoul National University College of Medicine, Seoul, 03080 Republic of Korea

## Abstract

The effect of aspirin on the risk of hepatocellular carcinoma (HCC) remains unclear. We investigated the association between aspirin use and HCC development in a region where viral hepatitis prevails. We conducted a population-based cohort study including a total of 460,755 participants who were tracked to identify incidents of HCC since 2007. The use of drug before the index date was assessed and standardized by the Defined Daily Dose system. We calculated the hazard ratios (HRs) and their 95% confidence intervals (CIs) for the association between aspirin use and HCC occurrence, using Cox proportional hazard regression models. There were 2,336 cases of HCC during the period of 2,965,500 person-years. Overall, aspirin users had a lower HCC risk (HR, 0.87; 95% CI, 0.77–0.98) than non-users in a dose-response manner (*P*_trend_ = 0.002). The protective effect of aspirin was amplified when combined with those of non-aspirin non-steroidal anti-inflammatory drugs (HR, 0.65; 95% CI, 0.50–0.85). Subgroup analyses revealed a significant chemopreventive effect of aspirin in individuals who were young, were male, or had viral hepatitis, whereas no protective effect was observed in patients with liver cirrhosis. Our results, suggesting different carcinogenic pathways between viral and non-viral etiologies, may validate the design of future intervention trials of aspirin for HCC prevention in eligible populations.

## Introduction

Hepatocellular carcinoma (HCC) is the sixth most commonly diagnosed cancer worldwide and its incidence is anticipated to increase further in the next decade^1^. HCC continues to lead to unsatisfactory patient outcomes, even after curative treatment, because of its aggressive growth and high rates of recurrence and metastasis, which cause it to be the second most common cause of cancer death^2^. Moreover, because early detection methods for HCC are ineffective, the implementation of preventive measures is of considerable importance^3^.

Chemoprevention to reduce the risk of HCC is greatly appealing. Universal hepatitis B-virus (HBV) vaccination has substantially reduced the risk of HBV-associated HCC by decreasing the rate of new HBV infections^4^. Furthermore, antiviral therapies are consistently effective in preventing or delaying HCC^5,6^; however, their limited efficacy, dose-limiting side effects, high costs, and vulnerability to the emergence of drug-resistant mutants restrict their widespread use. Hence, there is a pressing need for new drugs that reduce the risk of HCC. Several medications commonly prescribed in primary practice, such as aspirin, have recently gained attention as promising protectors against HCC^7^. These non-etiology-specific drugs are inexpensive, have favorable rates of adverse events, and might have several extra-hepatic benefits.

Aspirin, because of its antiplatelet effect due to cyclooxygenase (COX) inhibition, is frequently prescribed to decrease the risk of cardiovascular disease-related mortality. Because chronic inflammation plays a pivotal role in the pathogenesis of HCC, chemoprevention of HCC by aspirin has been suggested^8^. Besides the inflammatory process, platelets have effects on immune modulation in the liver, facilitating immune-mediated liver injury and carcinogenesis^9^. In a murine model of chronic hepatitis B, treatment with aspirin reduced hepatic inflammation, fibrosis, and HCC development^10^.

A few epidemiologic studies investigated the effect of aspirin on the primary prevention of HCC, but the results were inconsistent^11–13^. Those studies, specifically designed to address effects of aspirin on HCC prevention, had major flaws: (i) too few HCC cases^14,15^, (ii) the use of self-reporting to identify previous aspirin intake with only time point of exposure^12,13^, (iii) no confirmation of dose-dependent or duration-dependent effects^12^, and (iv) no consideration of potential confounders such as other putative agents (e.g., statin and metformin) and underlying liver disease (e.g., chronic viral hepatitis and/or liver cirrhosis)^12^.

HCC is prevalent in East Asia, including Korea, where chronic HBV infection is endemic. To clarify the relationship between aspirin use and HCC risk in Korea, we performed a cohort study of a high-risk population using nationwide pharmaceutical claims data.

## Results

### Demographic characteristics of the cohort

Of the 460,755 participants included in the final cohort, 14.1% used aspirin (≥30 Daily Defined Doses [DDDs]). Table 1 lists the demographics, medical conditions, and medication use of the study cohort stratified by aspirin use. The median age of the aspirin users and non-users was 58 and 49 years, respectively. Compared with the aspirin non-users, the aspirin users were more likely to have healthy habits (smoked or drankless and had more physical activity) but were generally at higher risk for HCC, because they were more obese, had higher blood pressure/serum glucose/serum cholesterol levels, and had more comorbidities.

**Table 1:** Characteristics of the study population by aspirin use.

Characteristic	All subjects (N = 460,755)		Aspirin user (n = 64,782)		Aspirin non-user (n = 395,973)	
	No.	%	No.	%	No.	%
Age, years						
40–49	222,481	48.29	14,428	22.27	208,053	52.54
50–59	129,866	28.19	21,402	33.04	108,464	27.39
60–69	84,481	18.34	21,830	33.70	62,651	15.82
≥70	23,927	5.19	7,122	10.99	16,805	4.24
Median (IQR)	50 (44–59)		58 (50–65)		49 (44–57)	
Sex						
Men	247,008	53.61	33,230	51.30	213,778	53.99
Women	213,747	46.39	31,552	48.70	182,195	46.01
Body mass index, kg/m^2^						
<18.5	10,913	2.37	888	1.37	10,025	2.53
18.5–22.9	161,184	34.98	15,635	24.13	145,549	36.76
23–24.9	127,696	27.71	17,627	27.21	110,069	27.80
25–29.9	147,825	32.08	27,173	41.95	120,652	30.47
≥30	13,137	2.85	3,459	5.34	9,678	2.44
Median(IQR)	23.9 (22.0–25.8)		24.8 (23.0–26.8)		23.7 (21.9–25.7)	
Smoking status						
Never	320,104	69.47	47,758	73.72	272,346	68.78
Former	39,990	8.68	5,780	8.92	34,210	8.64
Current	95,325	20.69	10,403	16.06	84,922	21.45
N/A	5,336	1.16	841	1.30	4,495	1.14
Alcohol consumption/week						
None	269,203	58.43	41,875	64.64	227,328	57.41
<1 drinks	65,886	14.30	7,443	11.49	58,443	14.76
1–2 drinks	74,640	16.20	8,688	13.41	65,952	16.66
≥3 drinks	48,300	10.48	6,318	9.75	41,982	10.60
N/A	2,726	0.59	458	0.71	2,268	0.57
Physical activity/week						
None	241,823	52.48	33,826	52.22	207,997	52.53
1–2	116,948	25.38	14,364	22.17	102,584	25.91
≥3	98,020	21.27	15,948	24.62	82,072	20.73
N/A	3,964	0.86	644	0.99	3,320	0.84
Blood pressure category*						
Normal	126,031	27.35	9,179	14.17	116,852	29.51
Prehypertension	207,200	44.97	26,785	41.35	180,415	45.56
Hypertension	127,524	27.68	28,818	44.48	98,706	24.93
Fasting plasma glucose, mg/dL						
<100	307,813	66.81	35,388	54.63	272,425	68.80
100–125.9	115,967	25.17	19,309	29.81	96,658	24.41
≥126	36,975	8.02	10,085	15.57	26,890	6.79
Total cholesterol, mg/dL						
<200	242,966	52.73	32,713	50.50	210,253	53.10
200–239	154,876	33.61	21,721	33.53	133,155	33.63
≥240	62,913	13.65	10,348	15.97	52,565	13.27
CCI^†^, mean (SD)	1.55 (1.47)		2.64 (1.79)		1.37 (1.33)	
Socioeconomic status						
Quartile1	139,040	30.18	20,103	31.03	118,937	30.04
Quartile2	116,893	25.37	16,097	24.85	100,796	25.46
Quartile3	123,034	26.70	16,311	25.18	106,723	26.95
Quartile4	81,788	17.75	12,271	18.94	69,517	17.56
Statin, DDD						
<30	421,235	91.42	45,361	70.02	375,874	94.92
30–365	31,701	6.88	14,363	22.17	17,338	4.38
>365	7,819	1.70	5,058	7.81	2,761	0.70
Median^‡^ (IQR)	140 (65–305)		183 (81–376)		108 (60–236)	
Metformin, DDD						
<30	439,872	95.47	55,471	85.63	384,401	97.08
30–365	13,948	3.03	6,055	9.35	7,893	1.99
>365	6,935	1.51	3,256	5.03	3,679	0.93
Median^‡^ (IQR)	233 (101–453)		249 (114–468)		221 (93–440)	
Non-aspirin NSAIDs, DDD						
<30	347,182	75.35	41,175	63.56	306,007	77.28
30–90	76,141	16.53	14,043	21.68	62,098	15.68
>90	37,432	8.12	9,564	14.76	27,868	7.04
Median^‡^ (IQR)	61 (41–115)		71 (44–146)		59 (40–108)	
Aspirin, DDD						
<30	395,973	85.94	—		395,973	100.00
30–365	31,188	6.77	31,188	48.14	—	
365–730	13,781	2.99	13,781	21.27	—	
≥730	19,813	4.30	19,813	30.58	—	
Median^‡^ (IQR)	390 (127–858)		390 (127–858)		—	

**Abbreviations:** IQR = interquartile range; CCI = Charlson comorbidity index; SD = standard deviation; DDD = defined daily dose; NSAIDs = non-steroidal anti-inflammatory drugs.

^*^Normal, SBP < 120 mmHg and DBP < 80 mmHg; prehypertension, 120 mmHg ≤ SBP < 140 mmHg or 80 mmHg ≤ DBP < 90 mmHg; hypertension, SBP ≥ 140 mmHg or DBP ≥ 90 mmHg.

^†^Including acute myocardial infarction, congestive heart failure, peripheral vascular disease, cerebral vascular accident, dementia, pulmonary disease, connective tissue disorder, peptic ulcer, liver disease, diabetes, diabetes complications, paraplegia, renal disease, severe liver disease, and HIV infection based on ICD-10 codes of hospital visits during years 2003 through 2006.

^‡^The median prescribed number of DDDs for every study drug used (≥30 DDDs) in the cohort.

### Aspirin use and HCC risk

There were 2,336 cases of HCC in the entire cohort during the observation period of 2,965,500 person-years; the overall incidence was 78.8 HCCs per 100,000 person-years (95% CI, 75.6–82.0). The HCC incidence was 76.2, 103.7, 88.1, and 87.5 among participants with aspirin use of <30, 30–365, 365–730, and ≥730 DDDs, respectively (Table 2). There was a dose-dependent relationship between aspirin use and the risk of HCC (*P* for trend = 0.002). The adjusted hazard ratios [HRs] (95% confidence intervals [CIs]) were 0.98 (0.84–1.15), 0.79 (0.62–1.00), and 0.75 (0.60–0.91) for individuals with aspirin use of 30–365, 365–730, and ≥730 DDDs, respectively. A propensity score matching analysis also revealed the similar result with original multivariate analysis (Supplemental Table 1). No difference in mortality between aspirin users and aspirin non-users was noted during the observation period (Supplemental Table 2).

**Table 2:** Incidence and HRs of HCC associated with aspirin use.

				Incidence rate of HCC			Risk* of HCC			
	No. of subjects	No. of person-years	No. of HCC	per 10^5^ person-years	95% CI		Adjusted HR	95% CI		*P* for trend
All subjects	460,755	2,965,500	2,336	78.77	75.64	82.03				
Women	213,747	1,394,642	697	49.98	46.40	53.83	1			
Men	247,008	1,570,858	1,639	104.34	99.41	109.51	**2.17**	**1.95**	**2.41**	
Aspirin use										
Non-user (<30 DDDs)	395,973	2,565,103	1,954	76.18	72.87	79.63	1			**0.002**
User (≥30 DDDs)	64,782	400,397	382	95.41	86.30	105.47	0.87	0.77	0.98	
30–365 DDDs	31,188	192,907	200	103.68	90.26	119.09	0.98	0.84	1.15	
365–730 DDDs	13,781	85,143	75	88.09	70.25	110.46	0.79	0.62	1.00	
>730 DDDs	19,813	122,346	107	87.46	72.36	105.70	**0.75**	**0.60**	**0.91**	
Concurrent non-aspirin NSAID use (≥30 DDDs)										
Neither	325,136	2,114,601	1667	78.83	75.14	82.71	1			
Aspirin only user (≥365 DDDs)	22,046	137,237	123	89.63	75.11	106.95	**0.76**	**0.62**	**0.92**	
Non-aspirin NSAID only user	102,025	643,410	487	75.69	69.26	82.72	**0.81**	**0.73**	**0.91**	
Both user	11,548	70,253	59	83.98	65.07	108.39	**0.65**	**0.50**	**0.85**	

**Abbreviations:** HR = hazard ratio; HCC = hepatocellular carcinoma; DDD = defined daily dose; NSAIDs = non-steroidal anti-inflammatory drugs; CI = confidence interval.

^*^Cox proportional hazards regression models were used to calculate the adjusted hazard rate ratios and two-sided 95% confidence intervals, with adjustment for age, sex, body mass index, health behaviors (cigarette smoking, alcohol consumption, and physical activity), concurrent medication, category of blood pressure, fasting plasma glucose and total cholesterol, socioeconomic status, and Charlson comorbidity index score.

To sight the potential contribution of the COX enzyme, we further analyzed the additive effects of aspirin use in combination with non-aspirin non-steroidal anti-inflammatory drug (NSAID) use. In this analysis, individuals who used more than 365 DDDs of aspirin were considered aspirin users, considering both the robust effect of aspirin and the sufficient number of HCC cases for analysis. Compared with participants who used neither aspirin nor any other NSAID, users of aspirin only (HR, 0.76; 95% CI, 0.62–0.92) or of non-aspirin NSAIDs only (HR, 0.81; 95% CI, 0.73–0.91) had a reduced risk of developing HCC. The risk of developing HCC was far lower among participants who used both aspirin and other NSAIDs (HR, 0.65; 95% CI, 0.50–0.85).

In the sensitivity analysis, the shifting of the index date had little effect on the association between aspirin use and the incidence of HCC (Table 3). The statistical significance might have depended on the follow-up duration, with a longer follow up more likely to result in statistical significance. Table 3 shows whether the effects of aspirin were significant when the data were stratified according to age, sex, and underlying liver disease. The protective effect of aspirin against HCC development was significant among males who had more than 365 DDDs of aspirin, participants younger than 60 years of age who had more than 365 DDDs of aspirin, and patients with viral hepatitis who had more than 730 DDDs of aspirin. Aspirin had no protective effect in patients with liver cirrhosis.

**Table 3:** Sensitivity analysis of adjusted* HRs of aspirin use for risk reduction of hepatocellular carcinoma.

	No. of HCC/total subjects	30–365 DDDs			365–730 DDDs			≥730 DDDs		
		HR	95% CI		HR	95% CI		HR	95% CI	
*Shifting the index date, year*										
2005	2,938/480,193	0.92	0.78	1.08	**0.75**	**0.59**	**0.96**	**0.54**	**0.38**	**0.75**
2007 (main)	2,336/460,755	0.98	0.84	1.15	0.79	0.62	1.00	**0.75**	**0.60**	**0.91**
2009	1,606/439,865	0.89	0.74	1.08	1.03	0.81	1.31	**0.78**	**0.64**	**0.94**
*Subgroup effect*										
Age, years										
<60	1,640/382,013	0.99	0.80	1.21	**0.63**	**0.44**	**0.91**	**0.66**	**0.48**	**0.90**
≥60	716/132,853	1.04	0.83	1.31	1.03	0.75	1.41	0.89	0.68	1.18
Sex										
Women	697/235,741	1.02	0.78	1.33	1.03	0.70	1.50	0.84	0.59	1.21
Men	1,639/279,125	0.97	0.80	1.17	**0.69**	**0.51**	**0.93**	**0.71**	**0.55**	**0.91**
Liver disease										
No liver disease	1,203/389,256	1.19	0.96	1.46	0.84	0.59	1.18	1.02	0.78	1.34
Viral hepatitis	773/31,528	0.68	0.51	0.91	0.76	0.50	1.16	**0.51**	**0.33**	**0.79**
Alcoholic or toxic liver disease	461/40,720	0.91	0.65	1.27	1.20	0.76	1.89	0.59	0.33	1.06
Liver cirrhosis or hepatic failure	490/10,174	0.88	0.64	1.21	0.71	0.43	1.18	0.78	0.50	1.20

**Abbreviations:** HR = hazard ratio; DDD = defined daily dose; CI = confidence interval.

^*^Adjustment for age, sex, body mass index, health behaviors (cigarette smoking, alcohol consumption, and physical activity), concurrent medication, blood pressure category, fasting plasma glucose category, total cholesterol category, socioeconomic status, and Charlson comorbidity index score.

## Discussion

To firmly establish the efficacy of aspirin against HCC, randomized controlled trials (RCTs) are needed. However, given the low incidence of HCC and the slow rate of progression to HCC in the general population, it would be logically and ethically questionable to perform an RCT focused primarily on that issue^7^. Therefore, a well-designed, population-based cohort study, especially of a high-risk population, might be the best alternatives to address the question. In this large study with substantial duration, we found a dose-dependent association between aspirin use and the reduction of HCC risk, which was robust in the general population and in subpopulations including individuals who are young, are male, or have a history of viral hepatitis. Within patients with liver cirrhosis, we did not found any protective effect of aspirin.

Our study has a number of strengths, besides being conducted with large-scale, nationwide data from an area with a high HCC incidence. First, we used data collected prospectively. To avoid a “survivor bias” or an “immortal time bias”, our follow up started at the same calendar date for aspirin users and non-users, and aspirin use was treated as a time-dependent variable in Cox proportional hazard ratio models. Further, to examine the effect of aspirin use after the index date, we performed sensitivity analyses with different time frames, which produced similar overall trends. Second, we used the DDD system together with data from the prescription database to reliably assess drug exposure. The DDD system has been validated by the World Organization Collaborating Center for Drug Statistics Methodology, the membership of which covers most of the world and represents a compromise among countries that recommend different indications and doses. In addition, we verified all hospital prescriptions based on visits to the pharmacy within 14 days of the date of issue, after which time the prescriptions would become invalid. Third, we directly investigated the association between aspirin use and HCC risk, stratified according to underlying liver disease.

The two previous cohort studies^12,13^ that reported a preventive effect of aspirin on HCC development used questionnaire-based information about self-reported aspirin use and were subject to recall bias. Those studies did not report a cumulative dose-response relationship; the HCC risk reduction was similar between patients who reported monthly aspirin use and those who reported daily aspirin use^12^. To complement that defect, Yang *et al*. conducted a nested case-control study using the prescription database from the UK’s Clinical Practice Research Datalink^11^. Possibly because of potential confounders such as underlying liver disease and the possible use of other drugs, they did not find any efficacy of aspirin for the reduction of HCC risk (HR, 1.11; 95% CI, 0.86–1.44)^11^. The discrepancy between our results and those of that study^11^ might be partially explained by the prevalence of chronic viral infection (6.8% in our study vs. 1.4% in the previous study). It is well known that chronic viral infection is the most crucial cause of HCC: the HCC risk is increased 5–100-fold with HBV infection and 15–20-fold with HCV infection^16^.

The mechanism by which aspirin reduces HCC risk is not well understood. Aspirin may, however, differentially impact the risks of viral and non-viral hepatocarcinogenesis. Our study documents differential protective effects of aspirin according to the underlying liver disease. In the immune-mediated inflammatory process resulting from chronic viral hepatitis, platelets facilitate liver injury by promoting the accumulation of CD8^+^ T cells^17^. Sitia *et al*. showed that aspirin reduced T-cell-mediated inflammation and HCC progression in an HBV transgenic mouse model of chronic immune-mediated liver disease; aspirin failed to produce the same protective effect in a non-immunologically mediated, toxin-induced model of HCC^10^. Seemingly in line with our results are those of recent secondary prevention studies. Aspirin use was associated with better outcomes among patients with HBV-related HCC^18^ but did not reduce the recurrence risk of all-cause HCC (HR, 0.82; 95% CI, 0.64–1.06)^19^. Taken together, our findings highlight the need for future clinical trials to evaluate the impact of aspirin in patients with chronic viral hepatitis.

The finding that aspirin had no protective effect in patients with liver cirrhosis was unexpected, because the majority of HCC cases involve underlying viral cirrhosis. Aspirin does not appear to be effective for all individuals who have a high risk of developing HCC, considering that aspirin had no protective effect in elderly people despite the explicitly higher risk for HCC in that subpopulation (adjusted HR per 10 years, 1.25; 95% CI, 1.20–1.32; data not shown). A functionally inefficient, virus-specific CD8^+^ T-cell response causes continuous, low-level hepatocellular injury, which promotes hepatocellular proliferation and exposure to inflammatory mutagens^20^. With the repair functions compromised, the repetitive inflammatory cycle of necrosis and regeneration is thought to trigger random genetic alterations, ultimately leading to HCC development^20^. That cycle may happen less, however, under uncompromised conditions (e.g., immunosenescence)^21^. In addition, the potential confounding of the data by the indication is of concern, but it would rather overestimate the association in this population. Because of the high risk of gastrointestinal bleeding, aspirin might be given less to patients with more progressed cirrhosis and HCC. Although a recent review pointed out that excessive bleeding in some patients with advanced cirrhosis might be a lesser risk factor than thrombosis^22^, from the clinical perspective, aspirin prescription for patients with advanced liver disease should be minimized because of its diminished protective effect against HCC.

The inhibition of COX enzyme, especially COX-2, has been suggested as another potential mechanism to explain the chemoprotective effect of aspirin^23^. A large body of basic evidence supports an inverse association of COX-2 inhibition with HCC development. Celecoxib, a selective COX-2 inhibitor, inhibited hepatocellular growth by potentiating apoptosis^24,25^, which is similar to the effects of conventional NSAIDs^26^. Epidemiologic studies of the association between the use of non-aspirin NSAIDs and the risk of HCC have been inconclusive, however^11–14^. In our study, subgroup analyses revealed that the combined use of non-aspirin NSAIDs and aspirin was related to greater reduction of HCC risk, which is comparable to the result of a study of HCC postoperative outcomes^18^. Those suggest that aspirin has a chemopreventive mechanism that is distinct from those of other NSAIDs (i.e., an immunologic effect through anti-platelet properties).

Our results should be interpreted with caution. Efforts to advance from observational studies into clinical trials sometimes fail to reproduce associations^27^. Our results do not necessarily signify that giving aspirin to patients will reduce their likelihood of developing HCC. A fundamental flaw in our observational study arises from the non-randomized allocation of aspirin. An imbalance in unobserved covariates between aspirin users and non-users could influence the results, although we fully adjusted statistically for all of the observed covariates. Individuals who consistently engage in beneficial activities (e.g., using aspirin daily to reduce cardiovascular disease) may have a number of characteristics distinguishing them from individuals who do not consistently engage in such activities. Additionally, only 65.3% of all eligible Koreans participate in national health examinations, which might limit the generalizability of our results because of selection bias. Another inherent limitation is that prescription databases do not contain information about over-the-counter (OTC) aspirin use. There is no evidence, however, of massive OTC purchases of aspirin in Korea, and one simulation study indicated that, under many circumstances, missed OTC exposure may not invalidate results based on prescription data^28^. Finally, we could not assess clinical information about the treatment and extent of liver disease (i.e., the stage, viral load, and mutation), which could influence the risk of HCC.

In conclusion, long-term aspirin use may reduce the risk of HCC in patients with viral hepatitis. Our study was conducted with a unique cohort located where chronic viral hepatitis is common, using a large, longitudinal database containing complete prescription pharmaceutical information and health-examination data. Future RCTs and additional experimental studies are needed to clarify the mechanism linking aspirin use and HCC.

## Methods

### Data source and study population

We collected data from the medical insurance claims and biennial health examinations of a standardized cohort sampled from the Korean National Health Insurance Corporation (NHIC) claims database, which were provided for research purposes by the Korean NHIC with strict confidentiality guidelines.

Since 1995 in South Korea, the NHIC, the single insurer of the Korean public health-insurance sector, has provided compulsory universal health insurance covering all forms of health care services, in which 97% of the population is obliged to enroll. Clinics and hospitals submit claims for patient care; including electronic resources with demographic information, diagnoses, procedures, and prescriptions; to be reimbursed for 70% of the total medical costs. NHIC also provides biennial health examinations, conducted by medical staff at local hospitals, to individuals over 40 years of age. The NHIC databases have been used for epidemiologic research in the past, and the information about prescription use, diagnoses, and hospitalizations is of high quality^29^.

From the NHIC records, we obtained the following information about individuals who were 40 years of age or older and went to the Korean national health-examination service at least once between January 1, 2002 and December 31, 2006 (N = 514,866): sex, date of birth, socioeconomic status based on average insurance premium per month, details on admissions and outpatient visits, and comorbid conditions. We also obtained information about patient prescriptions, including the names of drugs, dosage, duration, and total expenditure. We verified that patient actually received the drugs prescribed by cross checking pharmacy visits.

We selected the examination nearest to the index date (January 1, 2007) and extracted the following information: height, weight, blood pressure, self-reported health-related habits (tobacco use, alcohol consumption, and physical activity), and blood chemistries (serum glucose and total cholesterol). Blood chemistries were measured under fasting conditions using clinical laboratories with a standard procedure.

**Figure 1:**
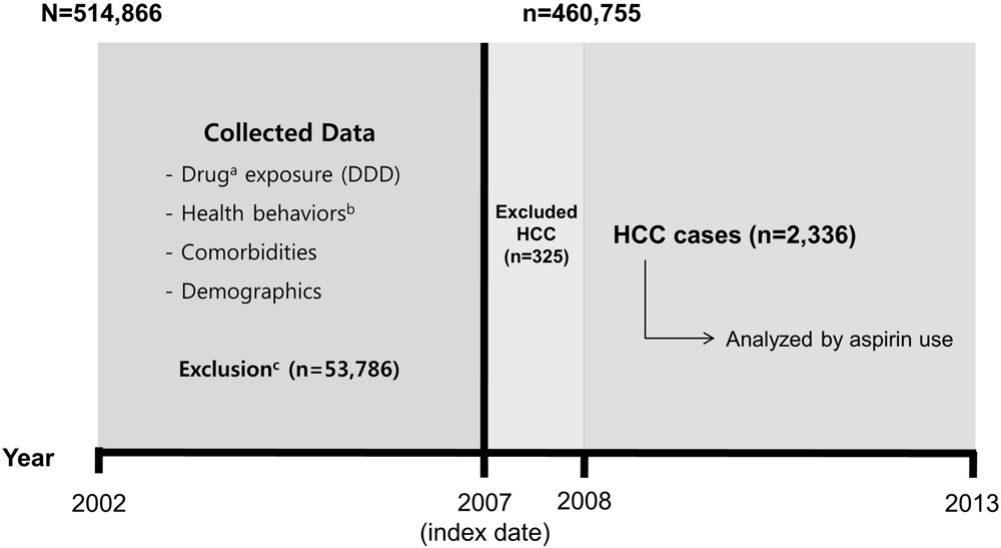
Study design and recruitment of participants. **Abbreviations:** DDD, defined daily dose; HCC, hepatocellular carcinoma; NHIC, National Health Insurance Corporation. ^a^Using the claims database of the NHIC, including non-steroidal anti-inflammatory drugs, statin, and metformin. ^b^From national health examinations, including smoking status, drinking habit, and physical activity. ^c^Patients with any cancer diagnosis with the ICD-10 “C” code, past medical history of cancer according to health-check survey data, or missing non-survey health check-up variables and those who died before the index date were excluded from the study.

We excluded from the study cohort individuals (n = 53,786) who had been diagnosed with any cancer, indicated by the *International Classification of Diseases code–10th Revision* (ICD-10) “C” code, had a medical history of cancer according to health check-up survey data, died before the index date, or were missing any non-survey health check-up variables. We allowed a latent period of 1 year, excluding HCC cases within 1 year after the index date (n = 325). Thus, we selected a total of 460,755 participants for the final analysis. The study design and recruitment of participants are depicted in Fig. 1.

The Institutional Review Board of Seoul National University Hospital (IRB number: E-1509–004–699) approved the study protocol. The ethics committee waived the need for participant consent, because the study involved routinely collected medical data that were anonymized at all stages, including during the data cleaning and statistical analysis. The methods were carried out in accordance with the relevant guidelines and regulations.

### Data collection

We observed the participants from the index date until December 31, 2013. The primary outcome was a newly diagnosis of HCC, represented by the ICD-10 code in the nationwide claims database. To reduce the effects of coding errors in the database, we defined HCC cases as those in which patients who visited the hospital at least once and received ICD-10 code “C220” met any of the following additional criteria: (i) made at least three outpatient visits associated with code “C220”, (ii) had a 3 or more days of admission with code “C220”, (iii) received radiation therapy, systemic chemotherapy, or therapy without chemotherapy or radiation claimed via Korean Diagnosis-Related Group (HDRG) code “H61-Malignacy of Liver”, (iv) died of causes related to code “C220”. In cases meeting those criteria, the first date of diagnosis under code “C220” was defined as the date of the event. The validity of using claims codes of the NHIC dataset for HCC as compared with the Korea Central Cancer Registry has been tested through comparing incidence rates. The accuracy of the HCC incidence in the NHIC dataset is above 95%^30^. Cases that met the criteria but involved a diagnosis of any other cancer prior to the date of the event were not considered HCC cases for the purpose of our analysis.

We extracted from the data all exposures and covariates recorded during the 5 years prior to the index date. The primary exposure of interest was the cumulative use of aspirin. We gathered data pertaining to aspirin prescriptions such as the date of the prescription, the daily dose, the number of days supplied, and the number of pills. We collected similar information about prescriptions for non-aspirin NSAIDs, statins, and metformin, which might influence the risk of HCC. To indicate the drug exposure, we used the DDD system, which assumes the average daily maintenance dose of an individual drug used for the drug’s main indication in adults. The DDD is a unit of measurement; it does not indicate a recommended dose or a real dose. We calculated the average daily dose (in units of DDD per day) of each prescription dispensed, weighted by the intended duration of each prescription. To examine the dose-response relationship, we categorized the cumulative aspirin use during the 5 years prior to the index date into four groups (<30, 30–365, 365–730, and >730 DDDs). We also categorized other drugs based on their median DDD, as appropriate. Patients who used less than 30 DDDs of a given drug were defined as non-users.

We expressed comorbid conditions as a Charlson comorbidity score, which was derived from ICD-10 codes in the claims database, using the sum of the weighted scores of all comorbidities (e.g., cardiovascular disease, pulmonary disease, renal disease, and liver disease, etc.)^31^.

We calculated body mass index (BMI) as the weight divided by the adult height squared (kg/m^2^). For analysis, we classified the participants into the following categories: BMI (<18.5, 18.6–22.9, 23–24.9, 25–29.9, or ≥30 kg/m^2^); blood pressure (normal, prehypertension, or hypertension); fasting glucose level (<100, 100–125.9, or ≥126 mg/dL or history of diabetes); cholesterol level (<200, 200–239, or ≥240 mg/dL or history of hyperlipidemia); frequency of physical activity (none, 1–2, or ≥3 times/week); smoking status (never, former, or current smoker); frequency of alcohol drinking (none, <1, 1–2, or ≥3 times/week).

### Statistical analysis

We calculated the incidence per 100,000 person-years by dividing the number of HCC events by the total number of person-years at risk and multiplying the result by 100,000. We calculated the 95% CI assuming a Poisson distribution.

We used Cox proportional hazards models to estimate the HRs and 95% CIs of HCC development with or without aspirin use. We calculated the accumulated person-years of risk, beginning with the index date and ending with the date of HCC diagnosis, diagnosis of any other cancer, death, or December 31, 2013, whichever came first—HCC diagnosis was the primary endpoint. To investigate the independent effect of aspirin on HCC risk, we conducted multivariable survival analyses using the Breslow method after adjusting for all potential confounders such as demographics, health risk behaviors, concurrent medications, and medical conditions. Furthermore, for a more unbiased result, we created a 1:1 propensity score matched cohort to compare the risk of HCC between aspirin users and aspirin non-users using the following covariates: age, sex, BMI, cigarette smoking, alcohol consumption, physical activity, blood pressure, fasting plasma glucose, Charlson comorbidity index, statin use, and metformin use.

Because drug consumption after the index date might confound the results, we performed a sensitivity analysis, shifting the index date forwards and backwards. We also conducted subgroup analyses stratified by age, sex, and underlying liver disease; in the stratified multivariable analyses, the association between aspirin use and the risk of HCC was reexamined in different subgroups. All analyses were performed using the STATA statistical software (version 11.0 for Windows; STATA Corp., Inc.). All statistical test results were considered statistically significant when two-tailed *p*-values were < 0.05.

## Electronic supplementary material

Supplemental Table 1–2
