# Supplementary material for: Aspirin Use and Risk of Hepatocellular Carcinoma in a National Cohort Study of Korean Adults
Source: Sci Rep. 2018 Mar 21;8:4968. doi: 10.1038/s41598-018-23343-0 (PMC5862896; doi:10.1038/s41598-018-23343-0)
Supplement: Supplementary file 1 — Supplemental Table 1–2 [file 41598_2018_23343_MOESM1_ESM.pdf]

Aspirin Use and Risk of Hepatocellular Carcinoma in a National Cohort Study of Korean Adults

In Cheol Hwang, Jooyoung Chang, Kyuwoong Kim, Sang Min Park

Supplemental Table 1

Risk of HCC in aspirin users compared with aspirin non-users in a 1:1 propensity score matched cohort*

	Aspirin non-users (<30 DDDs)	Aspirin users (≥30 DDDs)
No. of participants	75,248	75,248
No. of HCC	294	279
Person-years	214,945	237,776
HR (95% CI)	1 (reference)	0.86 (0.73–1.01)

Abbreviations: HCC = hepatocellular carcinoma; DDD = defined daily dose; HR = hazard ratio; CI = confidence interval.

*Matched for age, sex, body mass index, cigarette smoking, alcohol consumption, physical activity, blood pressure, fasting serum glucose, Charlson comorbidity index, statin use, and metformin use.

Supplemental Table 2

Risk of death in aspirin users compared with aspirin non-users

	Aspirin non-users (<30 DDDs)	Aspirin users (≥30 DDDs)
No. of participants	395,973	64,782
No. of death	15,891	5,827
Person-years	62,547	22,475
HR* (95% CI)	1 (reference)	0.99 (0.95–1.02)

Abbreviations: HCC = hepatocellular carcinoma; DDD = defined daily dose; HR = hazard ratio; CI = confidence interval.

*Adjusted for age, sex, body mass index, cigarette smoking, alcohol consumption, physical activity, blood pressure, fasting serum glucose, Charlson comorbidity index, statin use, and metformin use.
